# Bibliographical Department

**Published:** 1881-01

**Authors:** 


### BIBLIOGRAPHICAL DEPARTMENT.

"Nothing extenuate, nor set down aught in malice."

PRACTICAL TREATISE ON MATERIA MEDICA AND THERAPEUTICS.—By Roberts Bartholow, M. A., M. D., LL. D., Professor of

Materia Medica and General Therapeutics in the Jefferson Medical College, Philadelphia; recently Professor of the Practice of Medicine and of Clinical Medicine, and formerly Professor of Materia Medica and Therapeutics in the Ohio Medical College of Cincinnati; Author of a Manual of Hypodermic Medication; of the Russell Prize Essay on Quinine; of the American Medical Association Prize Essay on Atropia, and of the Fisk Fund Prize Essay on the Bromides, etc. Third Edition, revised. D. Appleton & Co., New York, 1880.

The rapidity with which this work has passed from one edition to another speaks more in its favor than any words we might write, yet we cannot withhold a few expressions in commendation of it. It is a purely American work and with the exception of that of Prof. H. C. Wood, is the only one in this country of any consequence that has attempted to present the Physiological action of remedies. The author is a gentleman of enthusiasm in his profession and an earnest and zealous worker in the field in which the foundation of this book has been laid, as far as possible; and while the plan and classification of remedial agents adopted are not invulnerable to criticism, they are probably about as good as almost any other that might have been attempted in our present state of knowledge of the subject. We have no doubt that the author will find this last edition more acceptable to the profession even than its predecessors.

A Treatise on the Practice of Medicnie, for the use of Students and Practitioners. By Roberts Bartholow, M. A., M. D., LL. D., Professor of Materia Medica and General Therapeutics in Jefferson Medical College of Philadelphia, formerly Professor of the Theory and Practice of Medicine and of Clinical Medicine in the Medical College of Ohio; Fellow of the College of Physicians of Philadelphia; Member of the American Philosophical Society; Honorary Member of the Medical and Chirurgical Faculty of Maryland; of the Ohio State Medical Society; of the Cincinnati Academy of Medicine; of the New York Neurological Society; President of the American Neurological Association, etc. D. Appleton & Co., New York, 1880.

No one will likely rise from a perusal of the above work without feeling he has been following the teaching of an expert, and though his enthusiasm should amount to eloquence sometimes, when speaking of the value of medicines, the book is none the less useful on that account.

We are glad to hear him speak with so much earnestness and confidence on this point, as *expectancy*, and great relying upon the *vis Medicatrix Naturæ* have found too much place in the minds of many members of the profession at the present time. More than a third of a century of active practice has increased our appreciation of the curative power of medicines, and to us, of course, the author's statements have the ring of true metal.

A careful examination of the volume cannot fail to interest even those who are not so enthusiastic on the subject as is Professor Bartholow, and it would doubtless tend greatly to inspire confidence in the minds of the most skeptical in our ranks should they venture to follow his lucid instructions in the treatment of even a very few cases. The terse and perspicuous style of the author, render the work very readable, and serve to present his views and opinions in such a manner as to make it acceptable to anyone. The book is not without its faults; notably so in the absence of general pathology of disease, so necessary in a work on practice. But they are rather those of omission, than commission, and seem to be almost inseparable from a new work on a subject, where the writer must necessarily record opinions and experiences of others as well as his own in catering for the general practitioner. We have no hesitancy, however, in recommending the work to the active physician as the best thus far produced on the subject in this country.

We are indebted to F. D. Lente, A. M., M. D., President of *The American Academy of Medicine*, for his valuable address before that body at its recent meeting in Providence, Rhode Island.

THE DETROIT FREE PRESS.—*A Weekly Publication;* (an exchange) its columns are filled with anecdote, paragraph and spicy comment, wit, humor, sketch and story; chess, puzzles, correspondence, sprightly editorials, travels, fashion, etc. "The Household," a weekly supplement, of interest to the ladies especially, is furnished gratis to every subscriber to *The Free Press.* Subscription $2.00 per annum. We can have it mailed to all $2.00 cash subscribers to THE INDEPENDENT PRACTITIONER for $1.25.

THE DENTAL DIRECTORY OF THE WORLD.—The compiler of this *work* still waits for more information before putting it to press. Deans of dental colleges, presidents of dental societies, and all who may have anything of importance to the dental profession, will please forward the same without delay. When possible, make all communications in the English language, printed or *plainly* written. Those having lists of dentists will confer a great favor by forwarding them. Dentists everywhere are earnestly requested to answer the following questions which are to them of personal importance:

1st. What is your full address, degrees, titles and honors?

2nd. What is your native tongue, and with what languages are you conversant?

3rd. From what college, and when did you take your degree?

4th. Of what societies are you a member?

5th. What office, if any have, you held?

6th. Will you subscribe (Price $5.00) for a copy of the book?

Please answer.

Address B. M. Wilkerson, M. D., D. D. S., 68 N. Charles street, Baltimore, Md., U. S. A.—Dental Journals please copy.

A CASE OF BREACH AND HEAD PRESENTATION.—A darkey who was stooping to wash his hands in a creek didn't notice the peculiar actions of a billy-goat just behind. So when he scrambled out of the water and was asked how it happened he answered: "I dunno 'xactly; but 'pears as if the sho kinder h'isted and frowd me."

THE following lines are by Benserade, and thus rendered by Dr. Johnson:

In bed we laugh, in bed we cry;
And born in bed, in bed we die.
The near approach, a bed may show,
Of human bliss to human woe.

